# Recent and Past Musical Activity Predicts Cognitive Aging Variability: Direct Comparison with General Lifestyle Activities

**DOI:** 10.3389/fnhum.2012.00198

**Published:** 2012-07-19

**Authors:** Brenda Hanna-Pladdy, Byron Gajewski

**Affiliations:** ^1^Departments of Neurology, and Radiology and Imaging Sciences, Emory University School of MedicineAtlanta, GA, USA; ^2^Departments of Biostatistics and Nursing, University of Kansas Medical CenterKansas City, KS, USA

**Keywords:** music, cognitive aging, modifiable factors of aging, lifestyle activities, training-induced changes

## Abstract

Studies evaluating the impact of modifiable lifestyle factors on cognition offer potential insights into sources of cognitive aging variability. Recently, we reported an association between extent of musical instrumental practice throughout the life span (greater than 10 years) on preserved cognitive functioning in advanced age. These findings raise the question of whether there are training-induced brain changes in musicians that can transfer to non-musical cognitive abilities to allow for compensation of age-related cognitive declines. However, because of the relationship between engagement in general lifestyle activities and preserved cognition, it remains unclear whether these findings are specifically driven by musical training or the types of individuals likely to engage in greater activities in general. The current study controlled for general activity level in evaluating cognition between musicians and nomusicians. Also, the timing of engagement (age of acquisition, past versus recent) was assessed in predictive models of successful cognitive aging. Seventy age and education matched older musicians (>10 years) and non-musicians (ages 59–80) were evaluated on neuropsychological tests and general lifestyle activities. Musicians scored higher on tests of phonemic fluency, verbal working memory, verbal immediate recall, visuospatial judgment, and motor dexterity, but did not differ in other general leisure activities. Partition analyses were conducted on significant cognitive measures to determine aspects of musical training predictive of enhanced cognition. The first partition analysis revealed education best predicted visuospatial functions in musicians, followed by recent musical engagement which offset low education. In the second partition analysis, early age of musical acquisition (<9 years) predicted enhanced verbal working memory in musicians, while analyses for other measures were not predictive. Recent and past musical activity, but not general lifestyle activities, predicted variability across both verbal and visuospatial domains in aging. These findings are suggestive of different use-dependent adaptation periods depending on cognitive domain. Furthermore, they imply that early age of musical acquisition, sustained and maintained during advanced age, may enhance cognitive functions and buffer age and education influences.

## Introduction

Cognitive aging variation is evident from studies documenting numerous individual characteristics associated with enhanced cognitive functioning in advanced age (Kramer et al., 2004). Age-related cognitive declines have consistently been documented primarily in reduced processing capacity and fluid abilities with acceleration in the fifth and sixth decades (Salthouse, 2004). Despite these declines, evidence suggests that measures of knowledge remain stable or improve with age and that there may be large individual variability in terms of successful cognitive aging (Anstey and Smith, 1999; Kramer et al., 2004). Thus, age-associated cognitive declines may not be inevitable, with increasing evidence that several factors and/or lifestyle activities may predict the course of cognitive development across the life span. Lifestyle factors are gaining support as modifiable variables in aging that may delay the expression of brain pathology theoretically because of greater ability to compensate for deficits through alternate neural mechanisms reflective of functional reserve (Cabeza et al., 2002; Scarmeas et al., 2003; Hall et al., 2009; Stern and Munn, 2010). Many positive environmental influences on cognition and brain plasticity during aging have been considered including physical and leisure activities, educational and occupational activities, bilingualism, and high levels of experience and expertise in either occupational or leisure pursuits (Kramer et al., 2004; Springer et al., 2005; Valenzuela and Sachdev, 2006; Bialystok et al., 2007; Craik et al., 2010). While maintaining cognitive vitality is critical for enhanced quality of life in advanced age, few human studies have systematically evaluated cognitive enrichment with most studies focusing on physical and leisure activities (Scarmeas et al., 2003; Verghese et al., 2003; Wilson et al., 2003). Animal and human data suggest that lifelong learning may contribute to cognitive vitality late in life by increasing synaptic complexity and neurogenesis, and that staying engaged in intellectually stimulating activities may protect and maintain cognitive and brain function (Greenough et al., 1986; Fillit et al., 2002; Kramer et al., 2004; Newson and Kemps, 2005; Green and Bavelier, 2008). Cognitively stimulating activities such as playing bridge, completing cross word puzzles, and high educational and occupational attainment are associated with better cognitive functioning in advanced age, but it is difficult to determine whether these are related to the cognitive aspects of activity-induced learning or related to the types of individuals likely to engage in greater activities either throughout their life or in advanced age (Kramer et al., 2004; Newson and Kemps, 2005). Also, quantification of cognitively stimulating activities across the life span is impractical given that individuals would be required to retrospectively estimate the number of hours spent reading, playing games, or completing cross word puzzles, making it difficult to discern the critical timing of engagement and durations needed for optimal outcomes.

Although most learning paradigms employed in the laboratory designed to facilitate cognitive enhancement are specific and poorly generalize to other tasks, several lines of recent evidence offer hope for transfer effects with more extensive training (Jaeggi et al., 2008; Karbach and Kray, 2009). Also, complex real life activities involving skilled movements such as musical training, video games, golf and juggling are more likely to yield general learning effects (Boyke et al., 2008; Forgeard et al., 2008; Green and Bavelier, 2008; Bezzola et al., 2011, 2012). Instrumental musical activities are cognitively and motorically complex, tapping into many systems in parallel (auditory, sensorimotor, visuospatial, memory, processing speed, working memory), and require intensive repetitive practice over many years that is likely to yield differential brain organization that has the potential to yield more robust transfer across tasks related to enhanced brain plasticity (Elbert et al., 1995; Gaser and Schlaug, 2003; Koelsch et al., 2005; Bangert et al., 2006; Fujioka et al., 2006; Green and Bavelier, 2008; Jancke, 2009a,b; Moreno et al., 2011a). Also, musical training can be readily quantified across the life span in terms of the number of years of practice, age of acquisition, and formal years of training, and therefore, may serve as an ideal model for quantifying the effects of cognitive stimulation throughout the life span on successful aging. There is a growing body of literature supporting the influence of musical training early in development in shaping non-musical cognitive and motor functions (Costa-Giomi et al., 2001; Ho et al., 2003; Schellenberg, 2004; Koelsch et al., 2005; Penhune et al., 2005; Schlaug et al., 2005; Fujioka et al., 2006; Moreno et al., 2011a). The strongest evidence of musical transfer to non-musical cognitive functions is derived from studies exploring the effect of musical training on speech and language (Loui et al., 2011; Ott et al., 2011; Patel, 2011; Shahin, 2011). However, with the focus on music education and development, few studies have evaluated how participation in musical activities may enhance cognition in advanced age.

In a recent study, we demonstrated that instrumental musicians with extended practice across the life span displayed better cognition in advanced age (60–83 years of age). Specifically, at least 10 years of musical participation across the life span had a strong predictive effect on preserved cognitive functioning across both verbal and visuospatial domains, and for executive processes (Hanna-Pladdy and Mackay, 2011). These cognitive advantages persisted even when the musicians were not active in advanced age, and were not accounted for on the basis of intelligence or education. This suggests that musical training may prove to be a modifiable factor that can enhance successful cognitive aging by increasing neuroplasticity, and is consistent with the range of cognitive advantages following musical training in children (Pantev et al., 2003; Forgeard et al., 2008; Moreno et al., 2011b). This is supported by a recent study that reported less age-related decline in central auditory processing for lifelong musicians (Zendel and Alain, 2012). While another study also identified auditory enhancements in instrumental musicians with extensive practice into middle adulthood (45–65 years of age), this study failed to reveal differences for visuospatial functions (Parbery-Clark et al., 2011). Moreover, this study did not replicate the association between extent of musical training or find significant contributions from age of acquisition, although methodological limitations such as verbal intelligence differences and inclusion of individuals with musical training in the non-musician group, may have obscured interpretation of the findings (Parbery-Clark et al., 2011). Previous work in middle-aged professional musicians revealed increased gray matter density in Broca’s area correlating with enhanced visuospatial functions suggesting that musicians may uniquely utilize a left lateralized network for visuospatial processing (Sluming et al., 2002, 2007). Furthermore, age-related volume reductions in frontal regions have demonstrated attenuation in atrophy for middle-aged professional musicians (Sluming et al., 2002). Therefore, based on recent findings, there is strong evidence supporting brain plasticity in lifelong musicians with potential transfer to non-musical cognitive functions.

Nonetheless, several questions remain including whether cognitive advantages in musicians are related to training effects or a selection factor of who engages in musical activity (i.e., more intelligent or more active individuals), and whether transfer to functions outside of the auditory/verbal domain is possible (Schellenberg and Peretz, 2008). Also, while we accounted for physical exercise in our first investigation, we did not account for general lifestyle activities making it unclear if increased general activity level in musicians may have accounted for differences between the groups instead of musical training (Stern and Munn, 2010). This is a plausible hypothesis given that general lifestyle activities have reliably predicted cognitive change in older adults (Newson and Kemps, 2005). Consequently, this warrants further investigation in particular related to whether musical training may buffer age-related cognitive declines in older individuals at the age of greatest risk for development of a neurodegenerative process (i.e., over the age of 60). In the current study, we selected a sample of subjects comparable to our first study to further ascertain whether general activity level between musicians and non-musicians might account for differences in cognitive outcomes. Based on our previous results, we only selected musicians with greater than 10 years of musical experience, since musicians with 1–9 years of training were not previously different from non-musicians (Hanna-Pladdy and Mackay, 2011). Second, we evaluated predictive models to try and identify whether there are critical aspects of musical experience such as timing of engagement (i.e., age of acquisition or continued activity in advanced age) that may predict cognitive aging variability. Although age of acquisition has been demonstrated as critical in acquiring language, few studies have directly compared the effects of past and more recent experience in determining how the timing of stimulation influences cognitive development across the life span. While some cognitive capacities such as language and related auditory/verbal functions may have early critical sensitive periods, other functions may be more amenable to cognitive stimulation later in life, informing us of the potential differences in plasticity that may be harnessed and guiding future models of cognitive stimulation.

## Materials and Methods

### Subjects

Seventy community-dwelling older adults between 59 and 80 years of age were selected for this study which was conducted at the Kansas University School of Medicine (KUMC). The following two groups of individuals were selected for the present study on the basis of their previous experience with instrumental musical participation across the lifespan: (1) *Non-musicians* (*n* = 37) – less than 1 year of musical participation, and (2) *Musicians* (*n* = 33) – more than 10 years of instrumental musical participation. Inclusion of musicians with greater than 10 years of experience was based on results from our previous study which demonstrated statistically significant cognitive differences between musicians with 10 or more years of experience relative to non-musicians, but no differences for musicians with 1–9 years of experience. In the current study, we selected an independent sample of subjects, but with similar characteristics to the previous study. The musician and non-musician groups were matched on age and education, were native English speakers and strongly right hand dominant as determined by the Edinburgh Handedness Inventory (at least +60 on the inventory; see Table 1; Oldfield, 1971). Subjects were non-demented based on neuropsychological and functional data (Adelaide Activities Profile) and did not endorse significant history of psychiatric, substance abuse, or chronic medical illness (Folstein et al., 1975; Clark and Bond, 1995; see Table 1). This study was approved by the KUMC institutional review board and written informed consent was obtained from all participants.

**Table 1:** **Means (SDs) for demographics and scaled scores for neuropsychological measures**.

	Non-musicians (*n* = 37)	Musicians (*n* = 33)	*F*	Sig. (*p* < 0.05)	Effect size
Age	68.81 (5.15)	68.45 (4.45)	0.095	0.759	0.001
Education	16.75 (1.75)	16.94(1.48)	0.219	0.641	0.003
AAP	44.25 (6.48)	45.33 (7.02)	0.445	0.507	0.007
Edinburgh inventory	87.76 (11.91)	91.06 (10.06)	1.55	0.217	0.022
WAIS-III information	12.58 (2.69)	13.21 (2.08)	1.16	0.285	0.017
D-KEFS semantic fluency	12.78 (3.38)	13.94 (3.05)	2.23	0.140	0.032
**D-KEFS letter fluency**	11.22 (3.15)	13.12 (3.49)	**5.76**	**0.019**	0.078*
D-KEFS switching fluency	12.46 (2.96)	13.00 (2.39)	0.694	0.408	0.010
Boston naming test	12.22 (2.85)	12.97 (2.36)	1.43	0.236	0.021
WAIS-III digit span	10.73 (2.30)	11.69 (3.07)	2.25	0.139	0.032
**WAIS-III LN sequencing**	11.37 (2.00)	12.36 (2.16)	**3.91**	**0.05**	0.054*
WMS-III spatial span	12.4 (3.04)	12.0 (2.69)	0.345	0.559	0.005
D-KEFS trails 1	12.45 (2.02)	12.15 (2.19)	0.373	0.543	0.005
D-KEFS trails 4	12.21 (1.70)	12.61 (1.48)	1.04	0.313	0.015
CVLT-II total (trials 1-4)	0.338 (0.951)	0.409 (0.852)	0.108	0.743	0.002
**CVLT-II SDFR**	0.203 (1.04)	0.636 (0.730)	**3.99**	**0.05**	0.055*
CVLT-II LDFR	0.270 (0.93)	0.470 (0.750)	0.957	0.331	0.014
WMS-III visual reproduction I	12.84 (2.78)	12.67 (2.41)	0.075	0.785	0.000
WMS-III visual reproduction II	15.19 (2.22)	14.82 (2.11)	0.509	0.478	0.004
ROCF copy	11.43 (1.44)	11.69 (1.19)	0.691	0.409	0.010
ROCF – immediate recall	11.57 (3.04)	11.52 (2.74)	0.006	0.940	0.000
ROCF – delayed recall	11.68 (2.71)	10.97 (3.04)	1.06	0.307	0.015
**Benton JLO**	54.24 (5.16)	56.51 (3.54)	**4.51**	**0.037**	0.062*
Benton visual form discrim.	31 (2.00)	39.97 (1.49)	0.005	0.943	0.000
WCST – perseverations	114.4 (23.5)	110.4 (22.1)	0.507	0.479	0.008
WCST – categories	3.22 (1.49)	3.00 (1.39)	0.390	0.535	0.006
Tower – total	11.92 (2.27)	11.79 (2.55)	0.052	0.820	0.001
**Tower – rule violation**	10.62 (0.72)	10.91 (0.290)	**4.57**	**0.036**	0.063*
Grooved pegboard-RH	7.62 (2.25)	8.69 (2.60)	3.43	0.068	0.048
Grooved pegboard-LH	7.41 (2.48)	8.45 (2.29)	3.36	0.071	0.047
Finger tapping-RH	7.86 (3.14)	8.55 (2.93)	0.874	0.353	0.013
Finger tapping-LH	7.67 (3.08)	8.89 (2.92)	2.79	0.100	0.039

*AAP, Adelaide activities profile; WAIS-III, Wechsler adult intelligence scale third edition; D-KEFS, Delis–Kaplan executive function system; CVLT-II, California verbal learning test second edition; SDFR, short delay free recall; LDFR, long delay free recall; WMS-III, Wechsler memory scale third edition; VR I, visual reproduction immediate recall; VR II, visual reproduction delayed recall; LNS, letter-number sequencing; JLO, judgment of line orientation; ROCF, Rey Osterrieth Complex Figure; WCST, Wisconsin card sorting task; RH, right hand; LH, left hand. AAP out of maximum 63; WCST Categories out of a maximum 6; JLO out of a maximum of 60 items; CVLT in *z* score deviations from the mean*.

*p < 0.05.

### Characteristics of musicians

The authors conducted a structured interview which was administered by the experimenter to obtain information regarding musical experience. The subjects were required to describe all musical experiences, age of acquisition, training settings, and exposure to various musical instruments and practice routines across their life span. Musicians selected for inclusion in the study were required to have a minimum of 10 years of musical activity with at least one musical instrument at any time in their life span. The majority of the musicians exceeded the minimum 10-year requirement (mean of 37 years), and 50% had experience with multiple instruments. The mean age of acquisition was 9.3 years of age, with a mean 4 years of formal musical training. Musicians were not required to be actively engaged in musical activities at the time of the evaluation, although close to half the group continued to actively participate in music with some regularity in advanced age. Piano was the most common instrument (61.8%), followed by strings (17.6%), horns (14.7%), woodwinds (2.9%) and percussion (2.9%). The characteristics of the musicians in the current study are similar to our previous study, with the exception that a greater proportion of high activity musicians (>10 years) in the current study had experience with multiple instruments (Hanna-Pladdy and Mackay, 2011).

### Level of general activity

We used the Adelaide Activities Profile (AAP) as a measure of general activity level (Clark and Bond, 1995). The AAP was developed from the Frenchay Activities Index, and is a validated measure of lifestyle activities in the elderly (Clark and Bond, 1995). The AAP provides a profile of the lifestyle activities of older adults by measuring behavior and physical capacity to carry out a number of daily tasks. On this scale, participants are asked to rate 21 items on a four-point Likert scale (scored between 0 and 3) to indicate their frequency of participation over the previous 3 months. Higher scores represent a higher frequency of participation in domestic, health, and social activities. Based on principal component analysis conducted by Clark and Bond, the AAP was grouped into four categories: household maintenance (e.g., gardening, car maintenance), domestic chores (e.g., washing dishes, preparing a meal), social activities (e.g., outdoor recreation or sports, participating in a club), and service to others (e.g., caring for other family members, doing volunteer work).

### Neuropsychological assessment

All participants received a comprehensive neuropsychological assessment similar to what is typically utilized in a clinical setting for evaluation of age-related cognitive declines. Neuropsychological evaluation is considered the most effective differential diagnostic method in discriminating pathophysiological dementia from age-related cognitive decline, and other related disorders (Grober et al., 1988; Morgan and Baade, 1997). While there are a number of different cognitive screening measures for age-related cognitive decline, they have demonstrated high rates of false negatives, and are not as sensitive. Consequently, full neuropsychological assessments are valuable as sensitive measures and provide more detailed assessment procedures for several cognitive domains, and to be able to discriminate normal aging from beginning dementia (Jacova et al., 2007). Since this study focuses on whether musical training may enhance successful cognitive aging related to neuroplasticity and cognitive reserve, we employed a clinical assessment that is sensitive to evaluation for the risk of the development of dementia (i.e., significant impairment in three cognitive domains with associated functional declines).

Measures from the following cognitive domains were included: memory, attention, language, visuospatial, executive, and sensorimotor functioning. See Table 1 for the specific measures included in the neuropsychological battery.

The information subtest of the WAIS-III was also administered, and provides a good estimate of general intellectual ability and verbal intelligence which is stable with advanced age (Wechsler, 1997a). Verbal memory performance was measured by the California Verbal Learning Test, Second edition (CVLT-II, standard version; Delis et al., 2000), while non-verbal memory was measured by the Wechsler Memory Scale Third Edition (WMS-III) Visual Reproduction I and II subtests (Wechsler, 1997b), and the Rey Osterrieth Complex Figure (ROCF; Rey and Osterrieth, 1939, 1993; Osterrieth, 1944). Verbal attention and working memory were measured by the Digit Span (DS) subtest of the WAIS-III, and the Letter-Number Sequencing (LNS) subtest of the WAIS-III (Wechsler, 1997a). Visual attention, working memory, and visuospatial functioning were measured by the Spatial Span (SS) subtests of the WMS-III (Wechsler, 1997b), Benton Judgment of Line Orientation (JLO), and Benton Visual Form Discrimination (BVFD; Benton et al., 1994). Delis–Kaplan Executive Function System (D-KEFS) Trails 1–5 which also measure cognitive flexibility by asking the subject to switch rapidly between numbers and letters (Delis et al., 2004). Verbal and language functions were measured with the Boston Naming Test (BNT; Kaplan et al., 1983), and D-KEFS letter and phonemic fluency (Delis et al., 2004). Frontal-executive functions were measured by the Wisconsin Card Sorting Test (WCST; Grant and Berg, 1948), and the D-KEFS Tower Test (Delis et al., 2004). The Finger Tapping Test (FT) was used to measure the speed of open loop movements, and required participants to place their hand on a finger tapping board and tap as fast as they could for five 10-s trials (Reitan and Wolfson, 1993). The Grooved Pegboard Test (GP) was used to assess closed loop movements for each hand, and required rotation of small grooved pegs and placement into a board filled with keyhole-shaped holes (Reitan and Wolfson, 1993).

### Statistical analyses

Several analyses of variance (ANOVA) were conducted on the neuropsychological measures to determine between-group differences based on musical activity across the lifespan (musicians versus non-musicians). We also fitted several different partition regressions, that partition data according to a non-parametric relationship between the independent variables and the dependent variables by creating a tree (SAS, 2008). A regression tree is a non-parametric model that makes no parametric assumption about the errors. For these reasons, it is not necessary to test parametric fulfillment. The process uses binary partitions. For each level of the tree, it splits into two parts. Regression trees are good for exploring relationships without having a prior model and the results are very interpretable (SAS, 2008). These regression trees estimate optimal cut-points of the independent variables that best predict a dependent variable (categorical or continuous). In order to avoid biased estimates of R square, a fivefold cross validation was reported. The regressions were conducted on the neuropsychological tests revealing between-group differences, to determine the predictors of cognitive performance in musicians.

## Results

### Group differences

#### Estimate of verbal intellectual ability

An ANOVA evaluating verbal intellectual ability did not reveal between-group differences for the Information subset of the WAIS-III, *F*(1, 68) = 1.16, *p* = ns (see Table 1 for means). Although the estimated verbal intellectual abilities of non-musicians were slightly lower than musicians, this was not statistically significant (Table 1).

#### Attention, working memory, and visuomotor integration

Between-subject effects were significant for verbal working memory as measured by the WAIS-III LNS subtest, *F*(1, 68) = 3.91, *p* < 0.05. Musicians (mean = 12.36) displayed higher scaled scores than non-musicians (mean = 11.37; see Table 1). ANOVAs for the Digit Span subtest of the WAIS-III, *F*(1, 68) = 2.25, *p* = ns, D-KEFS Trails 1, *F*(1, 68) = 0.373, *p* = ns, D-KEFS Trails 2, *F*(1, 68) = 0.003, *p* = ns, D-KEFS Trails 3, *F*(1, 68) = 0.058, *p* = ns, D-KEFS Trails 4, *F*(1, 68) = 1.035, *p* = ns, and D-KEFS Trails 5, *F*(1, 68) = 0.002, *p* = ns, were not significant between-groups for either verbal or visual attentional functions.

#### Language and fluency

D-KEFS letter fluency revealed significant between-group differences consistent with higher scaled scores for musicians (mean = 13.12) relative to non-musicians (mean = 11.22), *F*(1, 68) = 5.76, *p* < 0.05. There were no significant between-group differences for naming on the BNT, *F*(1, 68) = 1.43, *p* = ns, D-KEFS semantic fluency, *F*(1, 68) = 2.23, *p* = ns, or D-KEFS switching fluency, *F*(1, 68) = 0.694, *p* = ns.

#### Memory

Measures of verbal learning encoding on the CVLT-II were not significantly different for the total recall across the four trials, *F*(1, 68) = 0.108, *p* = ns. The short delay free recall of the CVLT-II revealed better performance for musicians relative to non-musicians, *F*(1, 69) = 3.99, *p* < 0.05 (see Table 1 for means), but no significant group differences for CVLT-II long delay free recall, *F*(1, 68) = 0.957, *p* = ns. The groups also did not differ on immediate non-verbal recall of the WMS-III Visual Reproduction test (VR I), *F*(1, 68) = 0.785, *p *= ns, or the delayed recall of the Visual Reproduction (VR II), *F*(1, 68) = 0.478 *p* = ns. There were no significant differences in non-verbal memory recall between the musicians and non-musicians on ROCF immediate recall, *F*(1, 68) = 0.006, *p* = ns, or ROCF delayed recall, *F*(1, 68) = 1.06, *p* = ns (Table 1).

#### Visuospatial

There were no significant differences in visuospatial constructions between the musicians and non-musicians on the ROCF copy, *F*(1, 68) = 0.691, *p* = ns, visuospatial working memory on the Spatial Span, *F*(1, 68) = 0.345, *p* = ns, or differences in capacity for complex visual form discrimination on the BVFD test, *F*(1, 68) = 0.005, *p* = ns. However, the musicians displayed better visuospatial judgment than the non-musicians on the JLO test, *F*(1, 68) = 4.51, *p* = 0.037 (see Table 1 for means).

#### Frontal-executive

On the WCST test, there were no significant differences between musicians and non-musicians in terms of number of perseverations, *F*(1, 68) = 0.507, *p* = ns, or total categories completed, *F*(1, 68) = 0.390, *p* = ns. On the D-KEFS Tower task, there were no differences for the total score, *F*(1, 68) = 0.052, *p* = ns, or movement accuracy, *F*(1, 68) = 0.106, *p* = ns, although the non-musicians committed more rule violations during planning compared to the musicians, *F*(1, 68) = 4.57, *p* < 0.05.

#### Sensorimotor

The GP test did not reveal significantly better performance on manual dexterity for musicians relative to non-musicians. However, a trend emerged revealing faster performance on the GP for musicians for both the right dominant, *F*(1, 68) = 3.43, *p* = 0.068, and the left non-dominant hands, *F*(1, 68) = 3.43, *p* = 0.071. There were no significant differences between musicians and non-musicians on finger tapping speed for either the dominant right hand, *F*(1, 68) = 0.874, *p* = ns, or non-dominant left hand, *F*(1, 68) = 2.79, *p* = ns (see Table 1 for means). However, additional analyses on motor measures with strength of handedness (Edinburgh Quotient) as a covariate were conducted. When controlling for strength of handedness, there was a significant Group effect for the GP for the dominant hand, *F*(1, 68) = 4.22, *p* = 0.044 and approaching significance for the non-dominant hand, *F*(1, 68) = 3.75, *p* = 0.057. Finger tapping speed remained insignificant bilaterally.

#### Active musical participation in advanced age

Since only half of the musicians remained musically engaged in advanced age, we evaluated differences between the currently active musicians and those who were inactive at the time of the evaluation (inactive, *n* = 16; active, *n* = 17). Active musicians did not differ significantly from inactive musicians in terms of age, years of education or activity level as measured by the AAP (see Table 2). They also did not differ significantly in terms of age of musical acquisition, *F*(1, 32) = 1.53, *p* = ns [mean (SD)_active_ = 8.41(2.89); mean (SD)_inactive_ = 10.19 (5.16)], or formal years of musical training, *F*(1, 32) = 2.86 *p* = ns [mean (SD)_active_ = 4.65 (2.98); mean (SD)_inactive_ = 3.31(1.08)]. Consistent with their continuation in musical activities in advanced age, active musicians devoted significantly more years to musical participation than inactive musicians *F*(1, 32) = 45.89 *p* < 0.001 [mean (SD)_active_ = 54.35 (16.15); mean_inactive_ = 18.56 (14.05)].

**Table 2:** **Means (SDs) scaled scores and significance for inactive and active musicians**.

	Inactive musicians (*n* = 16)	Active musicians (*n* = 17)	*F*	Sig. (*p* < 0.05)	Effect size
Age	67.50 (5.03)	69.35 (3.76)	1.45	0.238	0.047
Education	16.75 (1.48)	17.12 (1.49)	0.502	0.484	0.016
AAP	45.31 (5.99)	45.35 (8.05)	0.000	0.987	0.000
ROCF delay recall	10.06 (3.33)	11.82 (2.53)	2.94	0.096	**0.087**
D-KEFS letter fluency	12.13 (3.50)	14.06 (3.33)	2.65	0.114	**0.079**
WAIS-III LNS	11.94 (2.21)	12.77 (2.11)	1.21	0.279	0.038
CVLT-II SDFR	0.594 (0.757)	0.676 (0.737)	0.102	0.751	0.003
Benton JLO	55.81 (3.88)	57.17 (3.15)	1.24	0.275	0.038
Tower – rule violation	10.88 (0.342)	10.94 (0.242)	0.416	0.524	0.013

*AAP, Adelaide activities profile; WAIS-III, Wechsler adult intelligence scale third edition; D-KEFS, Delis–Kaplan executive function system; CVLT-II, California verbal learning test second edition; SDFR, short delay free recall; LNS, letter-number sequencing; JLO, judgment of line orientation; ROCF, Rey Osterrieth complex figure. AAP out of maximum 63; JLO out of a maximum of 60 items; CVLT in *z* score deviations from the mean*.

*p < 0.05*.

There were no significant differences between active and inactive musicians on verbal IQ estimates, neuropsychological measures, or for the specific measures that discriminated between non-musicians and musicians. However, a general trend emerged revealing better performance for active musicians relative to inactive musicians (see Table 2 for means and effect sizes). The delayed recall of the ROCF and D-KEFS letter fluency emerged with the largest effect sizes explaining 8.7% and 7.9% (Table 2) respectively, of the between subjects variance, with significance levels likely influenced by the small sample sizes.

#### Results of partition analyses of music data

The effect sizes for partition trees are summarized in Table 3, in order from smallest to largest cross-validated *R*^2^ (*f*^2^). Cross-validated versions of effect size avoid over fitting, common in non-parametric models (i.e. models not restricted to *linear* functions). Using Cohen’s (1988) convention of effect size *f*^2^, 0.02, 0.15, and 0.35 are *small*, *medium*, and *large*. Two of the partition trees evaluating neuropsychological measures of significance for the musicians only have medium effect sizes, and are highlighted below.

**Table 3:** **Effect sizes of all partition analyses**.

Dependent variable	Fivefolded cross-validated *R*^2^ (%)	Effect size, *f*^2^
**MUSICIANS ONLY**		
Judgment line orientation	15	0.18
Letter-number sequencing	14	0.16
Tower rule violations	0	0.00
Letter fluency	0	0.00
CVLT-II SDFR	0	0.00
Grooved pegboard – dominant hand	29	0.21
Grooved pegboard – non-dominant hand	28	0.20

**Figure 1:**
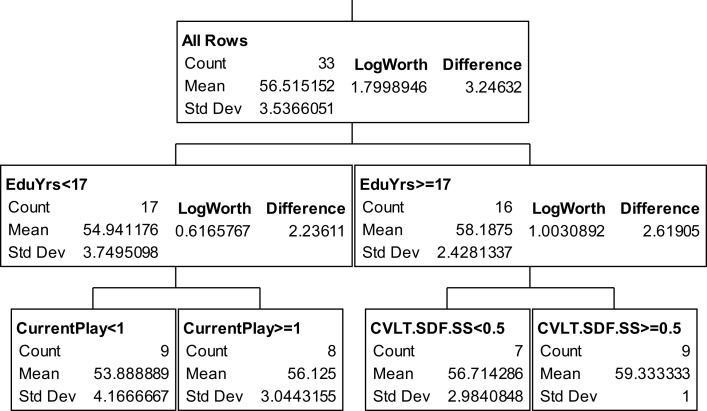
**Partition analysis judgment line orientation for musicians**.

**Figure 2:**
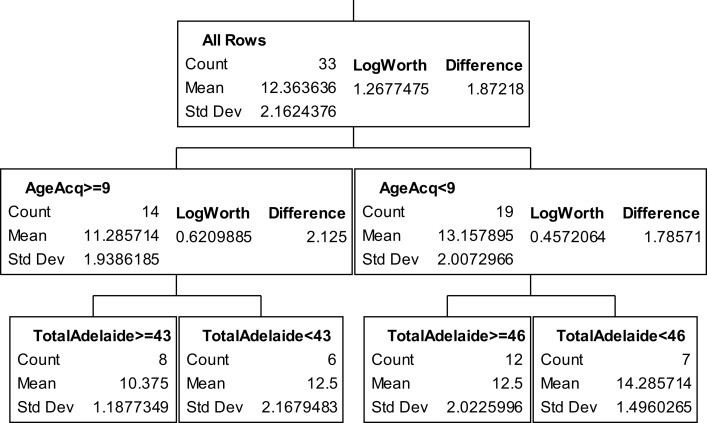
**Partition analysis for letter-number sequencing for musicians**.

**Figure 3:**
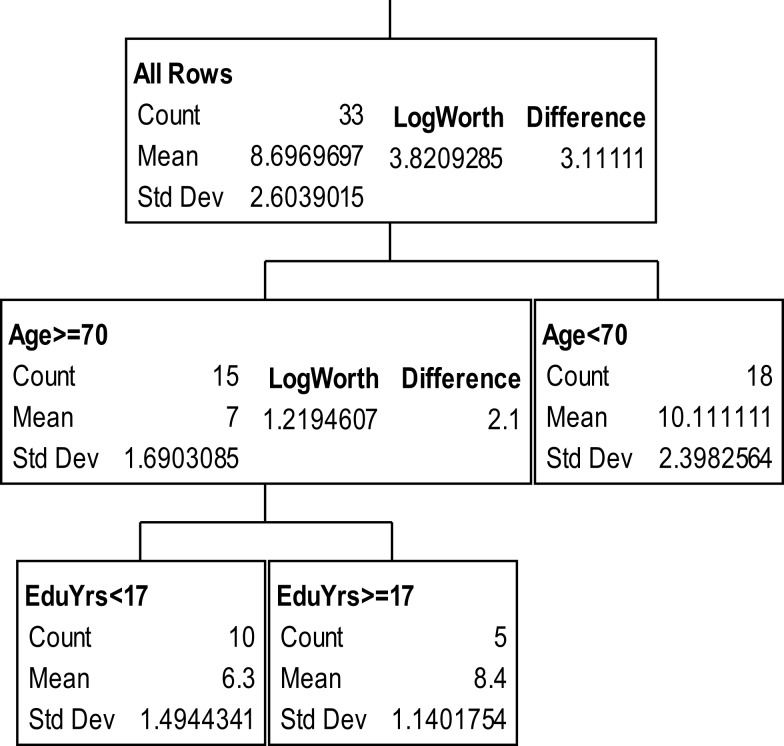
**Partition analysis for grooved pegboard dominant hand scaled scores**.

**Figure 4:**
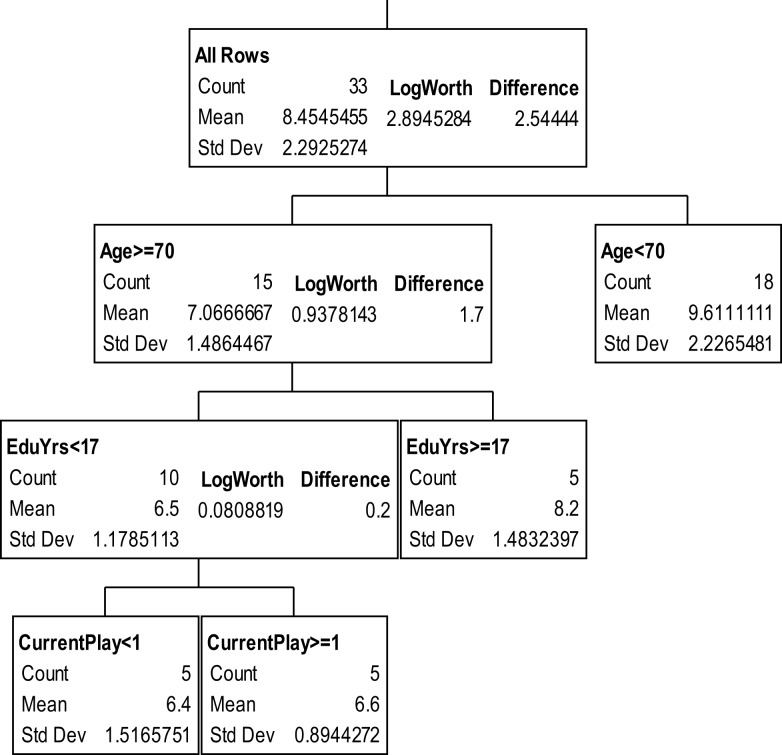
**Partition analysis for grooved pegboard non-dominant hand scaled scores**.

The partition trees for JLO (Figure 1), LNS (Figure 2), and GP dominant and non-dominant hands (Figures 3 and 4) demonstrated the largest effect sizes for the models of musicians with *f*^2^ = 0.18, 0.16, 0.21, and 0.20 respectively. Partition trees for CVLT-SDFR, Letter Fluency, and Tower were not significant for musicians. The musicians with higher education (i.e., greater than 17 years) had higher JLO scores (mean = 58.19, SD = 2.43) than the less educated musicians (mean = 54.94, SD = 3.75), while general activity level did not reliable predict JLO performance for the more educated. Among the less educated musicians, musicians with recent musical activity had higher scores (mean = 56.1, SD = 3.04) than those who did not actively play in advanced age (mean = 53.9, SD = 4.17). The LNS partition tree revealed that musicians with earlier age of acquisition (less than 9 years of age) had better verbal working memory functions (mean = 13.15, SD = 2.01) than musicians with age of acquisition after 9 years of age (mean = 11.28, SD = 1.94). Once again, general activity level did not reliably predict LNS performance. Among the older (>70 years of age) musicians, those with education greater than 17 years had higher GP dominant hand scores (mean = 8.4, SD = 1.14) than those with less than 17 years of education (mean = 6.3, SD = 1.5). However, among the older musicians with less than 17 years of education, active musical participation subtly enhanced non-dominant hand GP performance (mean = 6.6, SD = 0.894) relative to inactivity (mean = 6.4, SD = 1.52), although age less than 70 and education greater than 17 years was the best predictor of high GP performance.

## Discussion

The results of the current study reveal that older adults (59–80 years) who acquired music early in life and maintained musical activities for an extended period of time (minimum 10 years; mean 37 years), outperformed older control adults in non-musical cognitive domains of verbal working memory, verbal memory, verbal fluency, visuospatial, and planning functions. When accounting for strength of handedness, the musicians also outperformed non-musicians on sensorimotor integration. From an early age, musicians engage in intensive practice involving repetitive visual translation of musical notation into spatiotemporal aspects of skilled movements that create the sound of music. Thus, the cognitive domains enhanced in the amateur musicians in our study match the demands of musical skill acquisition and training. Similarly, voxel-by-voxel morphometry has revealed gray matter volume enhancements in motor, auditory, and visuospatial brain regions for musicians (Gaser and Schlaug, 2003).

Furthermore, the cognitive domains of significance in our current study overlap with our previously reported findings in an independent sample of aging adults (60–83 years of age), although specific tests administered were different and replication was not identical (Hanna-Pladdy and Mackay, 2011). The most striking difference between our two studies was the type of memory performance enhanced in musicians, and was partly related to inclusion of a more sensitive verbal memory test in the current study. While we did not find differences on verbal memory in the first study utilizing the short version of the CVLT-II, we did find higher verbal recall for musicians after a brief delay in the current study when utilizing the long CVLT-II which is a more sensitive test and also requires semantic organizational strategies. This result corresponds to previously reported differences in young musicians on a Korean version of this verbal memory test (Chan et al., 1998; Ho et al., 2003). In addition to verbal memory enhancement for older musicians, we also found differences in verbal fluency and verbal working memory functions. The overlap between language and verbal functions with musical networks has been given careful consideration especially given the auditory demands of music processing (Fujioka et al., 2006; Patel and Iversen, 2007; Forgeard et al., 2008).

Functional imaging results have revealed auditory-sensorimotor integration in musicians, whereby there is co-activation of a musical network whether musicians are passively processing auditory properties of music or providing the motor response (Lotze et al., 2003; Bangert et al., 2006). Consequently, it is not surprising that similar to our study results, there has been consistent demonstration of musical enhancement in auditory processing given the close link to musical cognitive demands (Pantev et al., 1998; Fujioka et al., 2006; Parbery-Clark et al., 2011). The neural basis of these enhancements are supported by large activations in the left hemisphere evident for musicians in prefrontal areas, supramarginal gyrus, and temporal areas varying depending on the musical cognitive processing requirements (Koelsch et al., 2005). These brain differences have been utilized as support for the presence of brain plasticity in longitudinal studies revealing differences in expected brain regions closely tied to musical skills, but also in brain regions unrelated to those skills responsible for multimodal integration (Hyde et al., 2009). These regions might possibly underlie the cognitive advantages in visuospatial processing identified in our study. At least one study has provided evidence suggesting that visuospatial advantages may be uniquely processed by a highly developed left hemisphere in musicians (Sluming et al., 2007). Although visuospatial advantages in musicians have not been consistently reported, there is a growing body of literature supporting non-verbal and visuospatial enhancements, but the underlying neural mechanisms are poorly understood (Costa-Giomi et al., 2001; Brochard et al., 2004; Forgeard et al., 2008).

Despite the obvious skilled movements associated with musical training, we did not find statistically significant differences between the groups for finger tapping speed which is in contrast to previously reported results (Jancke et al., 1997). It is conceivable that other age-related factors such as arthritis may have obscured significance in the motor domain, or perhaps the findings were not robust because musicians were not required to be musically active at the time of the study. This hypothesis is partially supported by the results of the partition analyses for sensorimotor functions. In addition to inactive musical participation in recent years, our participants differed from other studies in that they were all amateurs and therefore engaged in less extensive musical training which may have influenced the motor findings. Nonetheless, when controlling for strength of handedness, we did find differences in sensorimotor functions. This is consistent with evidence for expansion of cortical representations for musicians related to length of practice (Elbert et al., 1995). Also, gray matter differences between musicians and non-musicans has been identified extending from the premotor region to the primary somatosensory cortex into the anterior parietal lobe attributed to skill acquisition and practice (Gaser and Schlaug, 2003). Conversely, there is clear evidence that reduction of cortical representational areas accompanies reduced skilled use, in support of our less than robust motor findings for older adults with less recent activity (Liepert et al., 1995).

Despite group differences between musicians and non-musicians on a range of cognitive measures, partition analyses evaluating predictors of cognitive performance for the musical group only revealed significance on two tasks, JLO and LNS. These cognitive tasks span across both verbal and visuospatial domains, but both requiring fluid abilities. Similar to another recent study, we found verbal working memory but not spatial working memory differences and may be partially explained by differences in test sensitivity (Parbery-Clark et al., 2011). The finding that age of musical acquisition before age 9 predicts enhanced performance in verbal working memory functions in advanced age, supports the model of sensitive periods for auditory and language circuits. The maintenance of cognitive enhancements many years later irrespective of continued participation in musical activity, suggests that neural circuits during this critical period may be altered permanently. Indeed, increased size for the corpus callosum in musicians has been documented, but in particular in the anterior corpus callosum in the musicians who began musical training before the age of 7 (Schlaug et al., 1995). Consistent with our findings, this suggests that there is a maturation period within the first decade of life. However, since continued music participation predicted visuospatial functions, this raises the question of different sensitive periods for cognitive stimulation, or alternatively whether continued experience can alter connectivity patterns with the architectural constrains established during earlier sensitive periods (Knudsen, 2004). However, our results do not allow us to tease apart the reason for this association, and it is plausible that older individuals with enhanced cognitive sensory abilities in advanced age are more likely to persist with musical activity.

Education proved to have the greatest impact on performance in visuospatial judgment for musicians, although our results revealed that recent musical participation could compensate for lower educational levels (Caparelli-Daquer et al., 2009). These results imply that musical training may be considered an educational opportunity serving as additional cognitive stimulation outside of the traditional academic domain. Structural and functional changes in white matter, dorsolateral frontal, and inferior frontal regions offer strong support for the enhancements in frontal-networks functions (i.e., working memory, cognitive flexibility, and planning functions) for the musicians in our study (Hyde et al., 2009). Moreover, one longitudinal study with random assignment of young children into musical and non-musical groups, reported improvement in executive functions after only 20 days of musical training with additional neural evidence from corresponding ERP (Moreno et al., 2011a). It is conceivable that music training influences domain specific processes in verbal and auditory functions, but also domain-general processes such as attention and executive functioning (Hannon and Trainor, 2007). This hypothesis is supported by the results of our previous study which revealed that performance on a task requiring cognitive flexibility was the best cognitive predictor of musical status (Hanna-Pladdy and Mackay, 2011).

Many activities that are associated with cognitive stimulation may also increase social interactions and physical activity, making it difficult to discern whether it is the cognitive, social or physical aspect of the activity that is yielding the beneficial effect. Because of these challenges and the difficulty in randomly assigning subjects to musical and non-musical groups, there is a need to try and determine statistically whether musical effects are related to learning effects versus a population bias (i.e., highly educated individuals are more likely to engage in musical activities), or effect of increased general lifestyle activities (Schellenberg, 2004; Green and Bavelier, 2008). Our previous study accounted for the variance in cognitive aging variability related to physical exercise and demonstrated significant contributions to cognition from musical activity above that attributed to physical activity (Hanna-Pladdy and Mackay, 2011). Results of our partition analyses from the current study reveal that participation in general activities was not a reliable predictor of cognitive performance, and that musicians did not differ in general lifestyle activity engagement relative to non-musicians, making this hypothesis less plausible. However, there are several limitations which should be considered in interpretation of the current findings. First and foremost, while the current study controlled for general lifestyle activities, future studies will be needed to compare musical training to other specific leisure activities. Furthermore, given our small sample size, multiple comparisons is a limitation of this exploratory study especially since musicians only revealed statistical significance on five of the neuropsychological measures. Therefore, all results should be verified with prospective large studies with specific hypotheses generated based on our findings.

In summary, there is mounting evidence supporting training-induced brain changes from musical experience that can potentially transfer to non-musical cognitive abilities and influence cognitive functioning across the lifespan into advanced age (for review see Jancke, 2009a,b). However, further research is needed to fully understand the developmental mechanisms, and to tease apart the relative contributions from “nature and nurture” to musical skills and cognitive differences between musicians and non-musicians. By understanding differences in sensitive periods, and the range of activities that may stimulate cognition, we can gain deeper insight into the critical role that experience plays in shaping the brain across the lifespan. It remains unclear whether musical acquisition in adulthood affords any cognitive or neural advantages. Furthermore, longitudinal and neuroimaging studies of aging are needed to evaluate whether musicians may have enhanced cognitive reserve enabling them to better compensate for age-related cognitive declines, and reduce or delay the onset of cognitive decline or development of a neurodegenerative process.

## Conflict of Interest Statement

The authors declare that the research was conducted in the absence of any commercial or financial relationships that could be construed as a potential conflict of interest.
